# *catena*-Poly[[[aqua­(1,10-phenanthroline-κ^2^ *N*,*N*′)cadmium(II)]-μ-pyridine-2,3-dicarboxyl­ato-κ^4^ *N*,*O* ^2^:*O* ^3^,*O* ^3′^] dihydrate]

**DOI:** 10.1107/S1600536808037203

**Published:** 2008-11-20

**Authors:** Ming Li, Wuzu Ha, Liang Chang, Liangjie Yuan

**Affiliations:** a Department of Chemical Engineering, Wuhan University of Science and Engineering, Wuhan 430073, People’s Republic of China; b College of Chemistry and Molecular Science, Wuhan University, Wuhan 430072, People’s Republic of China

## Abstract

The title complex, {[Cd(C_7_H_3_NO_4_)(C_12_H_8_N_2_)(H_2_O)]·2H_2_O}_*n*_, is a one-dimensional coordination polymer, wherein the Cd atom is seven-coordinated by two 1,10-phenanthroline N atoms, one N and three O atoms from two different pyridine-2,3-dicarboxyl­ate ligands, and one water mol­ecule. It is further extended to a two-dimensional layer structure by hydrogen bonds and π–π stacking inter­actions [centroid-centroid distances of 3.560 (2) and 3.666 (2) Å]. There is a *C*4 water chain in the structure whose repeat unit contains four water mol­ecules with O⋯O distances in the range 2.748 (3)–2.795 (4) Å. One of the two H atoms of each water of hydration is statistically distributed over two positions with equal occupancy.

## Related literature

For potential applications of metal–organic coordination polymers, see: Moulton & Zaworotko (2001 ▶). For related structures, see: Gutschke *et al.* (1995 ▶); Li *et al.* (2006 ▶); Yu *et al.* (2004 ▶). For the structure of ice, see: Eisenberg & Kauzmann (1969 ▶).
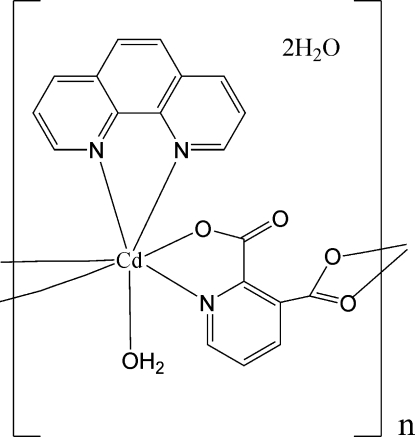


## Experimental

### 

#### Crystal data

[Cd(C_7_H_3_NO_4_)(C_12_H_8_N_2_)(H_2_O)]·2H_2_O
*M* *_r_* = 511.76
Triclinic, 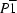

*a* = 7.8154 (5) Å
*b* = 10.5854 (7) Å
*c* = 13.0681 (8) Å
α = 70.934 (1)°
β = 77.940 (1)°
γ = 68.698 (1)°
*V* = 946.98 (10) Å^3^
*Z* = 2
Mo *K*α radiation
μ = 1.20 mm^−1^
*T* = 293 (2) K
0.40 × 0.16 × 0.15 mm

#### Data collection

Bruker SMART CCD area-detector diffractometer
Absorption correction: multi-scan (*SADABS*; Sheldrick, 1996 ▶) *T*_min_ = 0.645, *T*_max_ = 0.840
6124 measured reflections
4194 independent reflections
3979 reflections with *I* > 2σ(*I*)
*R*_int_ = 0.012

#### Refinement

*R*[*F* ^2^ > 2σ(*F* ^2^)] = 0.019
*wR*(*F* ^2^) = 0.048
*S* = 1.07
4194 reflections
272 parameters
8 restraints
H-atom parameters constrained
Δρ_max_ = 0.28 e Å^−3^
Δρ_min_ = −0.27 e Å^−3^

### 

Data collection: *SMART* (Bruker, 2001 ▶); cell refinement: *SAINT* (Bruker, 2001 ▶); data reduction: *SAINT*; program(s) used to solve structure: *SHELXS97* (Sheldrick, 2008 ▶); program(s) used to refine structure: *SHELXL97* (Sheldrick, 2008 ▶); molecular graphics: *ORTEP-3 for Windows* (Farrugia, 1997 ▶) and *DIAMOND* (Brandenburg, 1999 ▶); software used to prepare material for publication: *SHELXTL* (Sheldrick, 2008 ▶).

## Supplementary Material

Crystal structure: contains datablocks I, global. DOI: 10.1107/S1600536808037203/pv2111sup1.cif

Structure factors: contains datablocks I. DOI: 10.1107/S1600536808037203/pv2111Isup2.hkl

Additional supplementary materials:  crystallographic information; 3D view; checkCIF report

## Figures and Tables

**Table 1:** Hydrogen-bond geometry (Å, °)

*D*—H⋯*A*	*D*—H	H⋯*A*	*D*⋯*A*	*D*—H⋯*A*
O3*W*—H3*W*3⋯O2*W*	0.86	1.99	2.780 (3)	152
O3*W*—H2*W*3⋯O3*W*^i^	0.83	1.98	2.795 (4)	164
O3*W*—H1*W*3⋯O1^ii^	0.83	2.06	2.860 (2)	161
O2*W*—H1*W*2⋯O2^ii^	0.83	2.02	2.840 (2)	173
O2*W*—H3*W*2⋯O2*W*^iii^	0.84	1.93	2.748 (3)	163
O2*W*—H2*W*2⋯O3*W*	0.86	1.98	2.780 (3)	155
O1*W*—H2*W*1⋯O2^iv^	0.82	1.97	2.7784 (19)	168
O1*W*—H1*W*1⋯O3^v^	0.86	1.90	2.751 (2)	167

Symmetry codes: (i) 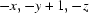; (ii) 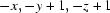; (iii) 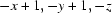; (iv) 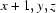; (v) 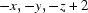.

**Fig. 1.**
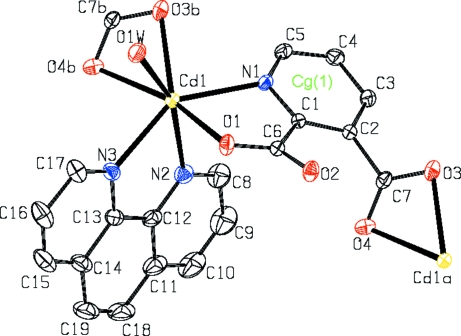
The coordination environment of Cd in (I) with the atom-labelling scheme. Displacement ellipsoids are drawn at the 30% probability level; hydrogen atoms were omitted for clarity. Symmetry codes: a = x - 1, y, z; b = x + 1, y, z.

**Fig. 2.**
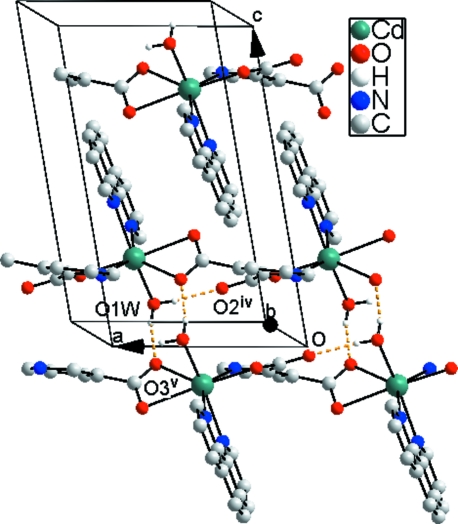
Unit cell packing of (I) showing (one-dimensional) chain-like structure along the a-axis; hydrogen bonds have been shown by dotted lines.

## supplementary crystallographic information

### Comment

Metal-organic coordination polymers have been of great interest due to their intriguing potential applications, such as catalysis, magnetism, electronic and chemical separation (Moulton & Zaworotko, 2001). Multidentate N– or O-donor ligands, such as pyridine- or imidazole- (di)carboxylic acids, have drawn extensive attention in the construction of coordination polymers or metal-organic formworks (MOF). For example, pyridine or imidazole dicarboxyic acid ligands, including pyridine-2,6-, 2,5- or 3,4-dicarboxylic and imidazole-3,4-dicarboxylic acids, have been extensively employed in the construction of such metal-organic formworks. Comparing with other pyridine-dicarboxylic acids, pyridine-2,3-dicarboxylic acid (2,3-pydc) has been rarely used as a linkage ligand (Gutschke *et al.*, 1995; Yu *et al.*, 2004; Li *et al.*, 2006). We have synthesized a novel one-dimensional (one-dimensional) coordination polymer based on 2,3-pydc, [Cd(2,3-pydc)(H_2_O)(phen).2H_2_O]_n _ (phen = 1,10-phenanthroline), (I), the crystal structure of which is presented in this article.

The title complex is a one-dimensional chain-like coordination polymer. In the structure of the title compound (Fig. 1), the Cd ion is seven-coordinated with two N atoms from phen, one N and three O atoms from two different pyridine-2,3-dicarboxylate and a water molecule. The 2,3-pydc affords four coordination atoms to connect two Cd ions, one as chelating bidentate through the N atom and one O atom of carboxylate in *2-*position, the other with two O atoms of carboxylate in *3-*position. Thus, complex (I) illustrates a one-dimensional chain structure along *a* axis, as shown in Fig. 2. Two adjacent chains band together by a series of hydrogen bonds involving water and carbonyl O-atoms (details are given in Table 1), π-π interaction of 1,10-phenanthroline with the shortest distance between the centroids of C11—C14/C18/C19 rings being 3.560 (2) Å and the shortest distance between the centroids of N3/C13—C17 rings are 3.666 (2) Å, thus resulting in a two-dimensional supramolecular structure. The structure also displays a short C6—O2···π(*Cg*(1)) interaction with a perpendicular distance between O2 and the centroid of *Cg*(1) being 3.562 (2) Å.

It is also worthwhile to note that there is a C4 water chain in (I), whose repeating unit contains four water molecules with O—O distances 2.750 (4) 2.782 (3), and 2.798 (4) Å (average distance = 2.777 Å), which are all close to the corresponding distance of O—O in the ice I_c_ (2.75 Å) and I_h_ (2.759 Å) determined at 143 and 183 K, respectively (Eisenberg & Kauzmann, 1969). Moreover, each water molecule links to the host by the H-bonding interaction between water of hydration and coordination water molecules. Water molecule can participate in four hydrogen bonds in a tetrahedral arrangement with two hydrogen atoms and two lone pairs, but also frequently show 3-coordinate configurations, just as in (I).

### Experimental

CdO (0.05 mmol), 1,10-phenanthroline (0.05 mmol) and pyridine 2,3-dicarboxylic acid (0.10 mmol) were added into 1 ml water and stirred for 5 min in air, then transferred to a closed container. After reacting at 353 K for 7 days, the mixture was cooled to room temperature at a rate of 5 K/h. Colorless crystals suitable for X-ray analysis were obtained.

### Refinement

All H atoms attached to C atoms of were fixed geometrically and treated as riding with C—H = 0.93 Å with *U*_iso_(H) = 1.5*U*_eq_(parent atom). Hydrogen atoms of water molecules were located in difference Fourier maps and included in the subsequent refinement using restraints (O—H = 0.85 (1) Å) with *U*_iso_(H) = 1.5*U*_eq_(O). The two hydrogen atoms were statistically distributed over two positions each (H2W2 and H3W2, H2W3 and H3W3) with occupation factors of 0.50.

### Crystal data

**Table:**

[Cd(C_7_H_3_NO_4_)(C_12_H_8_N_2_)(H_2_O)]·2H_2_O	*Z* = 2
*M**_r_* = 511.76	*F*_000_ = 512
Triclinic, *P*1	*D*_x_ = 1.795 Mg m^−^^3^
Hall symbol: -P 1	Mo *K*α radiation λ = 0.71073 Å
*a* = 7.8154 (5) Å	Cell parameters from 4951 reflections
*b* = 10.5854 (7) Å	θ = 2.3–29.6º
*c* = 13.0681 (8) Å	µ = 1.20 mm^−^^1^
α = 70.934 (1)º	*T* = 293 (2) K
β = 77.940 (1)º	Rod-like, colorless
γ = 68.698 (1)º	0.40 × 0.16 × 0.15 mm
*V* = 946.98 (10) Å^3^	

### Data collection

**Table:**

Bruker SMART CCD area-detector diffractometer	4194 independent reflections
Radiation source: fine-focus sealed tube	3979 reflections with *I* > 2σ(*I*)
Monochromator: graphite	*R*_int_ = 0.012
*T* = 293(2) K	θ_max_ = 27.5º
φ and ω scans	θ_min_ = 2.9º
Absorption correction: multi-scan(SADABS; Sheldrick, 1996)	*h* = −10→10
*T*_min_ = 0.645, *T*_max_ = 0.840	*k* = −13→13
6124 measured reflections	*l* = −16→14

### Refinement

**Table:**

Refinement on *F*^2^	Secondary atom site location: difference Fourier map
Least-squares matrix: full	Hydrogen site location: inferred from neighbouring sites
*R*[*F*^2^ > 2σ(*F*^2^)] = 0.019	H-atom parameters constrained
*wR*(*F*^2^) = 0.048	*w* = 1/[σ^2^(*F*_o_^2^) + (0.0181*P*)^2^ + 0.4298*P*] where *P* = (*F*_o_^2^ + 2*F*_c_^2^)/3
*S* = 1.08	(Δ/σ)_max_ = 0.001
4194 reflections	Δρ_max_ = 0.28 e Å^−^^3^
272 parameters	Δρ_min_ = −0.26 e Å^−^^3^
8 restraints	Extinction correction: SHELXL97 (Sheldrick, 2008), Fc^*^=kFc[1+0.001xFc^2^λ^3^/sin(2θ)]^-1/4^
Primary atom site location: structure-invariant direct methods	Extinction coefficient: 0.0051 (5)

### Special details

**Table:**

Experimental. Elemental analysis. Cacld. for C_19_H_17_CdN_3_O_7_: C, 44.55; H, 3.35; N, 8.21; Found: C, 44.05; H, 3.44; N, 8.53.
Geometry. All e.s.d.'s (except the e.s.d. in the dihedral angle between two l.s. planes) are estimated using the full covariance matrix. The cell e.s.d.'s are taken into account individually in the estimation of e.s.d.'s in distances, angles and torsion angles; correlations between e.s.d.'s in cell parameters are only used when they are defined by crystal symmetry. An approximate (isotropic) treatment of cell e.s.d.'s is used for estimating e.s.d.'s involving l.s. planes.
Refinement. Refinement of *F*^2^ against ALL reflections. The weighted *R*-factor *wR* and goodness of fit *S* are based on *F*^2^, conventional *R*-factors *R* are based on *F*, with *F* set to zero for negative *F*^2^. The threshold expression of *F*^2^ > σ(*F*^2^) is used only for calculating *R*-factors(gt) *etc*. and is not relevant to the choice of reflections for refinement. *R*-factors based on *F*^2^ are statistically about twice as large as those based on *F*, and *R*- factors based on ALL data will be even larger.

### Fractional atomic coordinates and isotropic or equivalent isotropic displacement parameters (Å^2^)

**Table:**

	*x*	*y*	*z*	*U*_iso_*/*U*_eq_	Occ. (<1)
Cd1	0.351199 (16)	0.150242 (13)	0.791379 (10)	0.02558 (5)	
O1	0.03910 (18)	0.25289 (13)	0.83701 (12)	0.0341 (3)	
O1W	0.3973 (2)	0.23034 (15)	0.92673 (11)	0.0386 (3)	
H1W1	0.3765	0.1750	0.9897	0.046*	
H2W1	0.5091	0.2167	0.9178	0.046*	
O2	−0.23661 (17)	0.22382 (14)	0.87797 (12)	0.0347 (3)	
O3	−0.39081 (19)	−0.04132 (16)	0.87081 (13)	0.0444 (4)	
O4	−0.3121 (2)	0.10668 (15)	0.72263 (12)	0.0431 (3)	
N1	0.2029 (2)	−0.02383 (15)	0.85619 (12)	0.0271 (3)	
N2	0.2943 (2)	0.15794 (18)	0.61441 (14)	0.0370 (4)	
N3	0.3212 (2)	0.37684 (16)	0.67259 (13)	0.0325 (3)	
C1	0.0212 (2)	0.03040 (17)	0.84658 (13)	0.0228 (3)	
C2	−0.0784 (2)	−0.04976 (17)	0.83637 (13)	0.0242 (3)	
C3	0.0120 (3)	−0.19266 (19)	0.84791 (15)	0.0311 (4)	
H3	−0.0519	−0.2500	0.8448	0.037*	
C4	0.1965 (3)	−0.24943 (19)	0.86391 (16)	0.0336 (4)	
H4	0.2575	−0.3455	0.8740	0.040*	
C5	0.2886 (3)	−0.16083 (19)	0.86460 (16)	0.0319 (4)	
H5	0.4147	−0.1976	0.8711	0.038*	
C6	−0.0676 (2)	0.18204 (17)	0.85371 (13)	0.0240 (3)	
C7	−0.2739 (2)	0.01160 (19)	0.80796 (15)	0.0286 (4)	
C8	0.2799 (4)	0.0538 (3)	0.5853 (2)	0.0539 (6)	
H8	0.2953	−0.0333	0.6367	0.065*	
C9	0.2429 (4)	0.0683 (4)	0.4814 (2)	0.0711 (8)	
H9	0.2360	−0.0079	0.4638	0.085*	
C10	0.2171 (4)	0.1952 (4)	0.4069 (2)	0.0715 (9)	
H10	0.1922	0.2067	0.3373	0.086*	
C11	0.2277 (3)	0.3097 (3)	0.43373 (18)	0.0540 (6)	
C12	0.2682 (3)	0.2858 (2)	0.54009 (15)	0.0371 (4)	
C13	0.2814 (3)	0.4003 (2)	0.57072 (15)	0.0353 (4)	
C14	0.2530 (3)	0.5338 (2)	0.49409 (18)	0.0483 (6)	
C15	0.2690 (4)	0.6423 (2)	0.5264 (2)	0.0581 (7)	
H15	0.2527	0.7313	0.4776	0.070*	
C16	0.3084 (4)	0.6176 (2)	0.6286 (2)	0.0572 (7)	
H16	0.3187	0.6892	0.6509	0.069*	
C17	0.3331 (3)	0.4828 (2)	0.70002 (19)	0.0446 (5)	
H17	0.3592	0.4667	0.7703	0.054*	
C18	0.1968 (4)	0.4480 (4)	0.3597 (2)	0.0708 (9)	
H18	0.1669	0.4643	0.2903	0.085*	
C19	0.2102 (4)	0.5535 (4)	0.3882 (2)	0.0674 (8)	
H19	0.1912	0.6418	0.3380	0.081*	
O2W	0.3981 (2)	0.49790 (17)	0.09955 (14)	0.0559 (4)	
H2W2	0.3059	0.4845	0.0842	0.067*	0.50
H1W2	0.3589	0.5781	0.1080	0.067*	
H3W2	0.4562	0.5171	0.0376	0.067*	0.50
O3W	0.0437 (3)	0.49496 (17)	0.09995 (15)	0.0593 (5)	
H1W3	−0.0028	0.5736	0.1119	0.071*	
H2W3	−0.0031	0.4996	0.0465	0.071*	0.50
H3W3	0.1599	0.4814	0.0833	0.071*	0.50

### Atomic displacement parameters (Å^2^)

**Table:**

	*U*^11^	*U*^22^	*U*^33^	*U*^12^	*U*^13^	*U*^23^
Cd1	0.02151 (8)	0.02702 (8)	0.02817 (8)	−0.00974 (5)	−0.00549 (5)	−0.00348 (5)
O1	0.0248 (7)	0.0268 (6)	0.0536 (8)	−0.0105 (5)	−0.0005 (6)	−0.0146 (6)
O1W	0.0330 (7)	0.0508 (8)	0.0362 (7)	−0.0193 (6)	−0.0036 (6)	−0.0105 (6)
O2	0.0216 (7)	0.0326 (7)	0.0505 (8)	−0.0084 (5)	0.0026 (6)	−0.0164 (6)
O3	0.0263 (7)	0.0505 (9)	0.0554 (9)	−0.0196 (7)	−0.0050 (6)	−0.0044 (7)
O4	0.0384 (8)	0.0416 (8)	0.0443 (8)	−0.0102 (7)	−0.0183 (6)	0.0002 (6)
N1	0.0205 (7)	0.0260 (7)	0.0348 (8)	−0.0076 (6)	−0.0066 (6)	−0.0058 (6)
N2	0.0356 (9)	0.0441 (9)	0.0339 (9)	−0.0136 (7)	−0.0041 (7)	−0.0127 (7)
N3	0.0307 (8)	0.0316 (8)	0.0301 (8)	−0.0100 (7)	0.0001 (6)	−0.0041 (6)
C1	0.0217 (8)	0.0235 (8)	0.0225 (8)	−0.0084 (6)	−0.0024 (6)	−0.0037 (6)
C2	0.0229 (8)	0.0242 (8)	0.0252 (8)	−0.0087 (7)	−0.0041 (6)	−0.0040 (6)
C3	0.0324 (10)	0.0255 (8)	0.0387 (10)	−0.0129 (7)	−0.0085 (8)	−0.0057 (7)
C4	0.0336 (10)	0.0225 (8)	0.0405 (10)	−0.0038 (7)	−0.0093 (8)	−0.0058 (7)
C5	0.0233 (9)	0.0284 (9)	0.0404 (10)	−0.0038 (7)	−0.0097 (7)	−0.0057 (8)
C6	0.0240 (8)	0.0245 (8)	0.0243 (8)	−0.0090 (7)	−0.0039 (6)	−0.0053 (6)
C7	0.0241 (9)	0.0285 (9)	0.0380 (10)	−0.0088 (7)	−0.0069 (7)	−0.0126 (7)
C8	0.0603 (16)	0.0619 (15)	0.0513 (14)	−0.0235 (13)	−0.0057 (11)	−0.0264 (12)
C9	0.078 (2)	0.097 (2)	0.0637 (18)	−0.0357 (18)	−0.0071 (15)	−0.0462 (18)
C10	0.0657 (18)	0.123 (3)	0.0417 (14)	−0.0371 (18)	−0.0068 (13)	−0.0351 (17)
C11	0.0397 (13)	0.0903 (19)	0.0300 (11)	−0.0203 (12)	−0.0050 (9)	−0.0136 (12)
C12	0.0250 (9)	0.0547 (12)	0.0268 (9)	−0.0103 (9)	−0.0025 (7)	−0.0077 (8)
C13	0.0230 (9)	0.0405 (10)	0.0294 (9)	−0.0057 (8)	0.0011 (7)	−0.0003 (8)
C14	0.0336 (11)	0.0488 (13)	0.0380 (11)	−0.0065 (10)	0.0012 (9)	0.0092 (9)
C15	0.0528 (15)	0.0359 (12)	0.0580 (15)	−0.0093 (11)	0.0093 (12)	0.0082 (10)
C16	0.0682 (17)	0.0346 (11)	0.0593 (15)	−0.0195 (11)	0.0141 (13)	−0.0098 (11)
C17	0.0519 (14)	0.0382 (11)	0.0418 (12)	−0.0191 (10)	0.0054 (10)	−0.0093 (9)
C18	0.0593 (17)	0.111 (3)	0.0266 (11)	−0.0262 (17)	−0.0137 (11)	0.0059 (14)
C19	0.0535 (16)	0.080 (2)	0.0391 (13)	−0.0158 (14)	−0.0086 (11)	0.0176 (13)
O2W	0.0545 (10)	0.0439 (9)	0.0604 (11)	−0.0030 (8)	−0.0024 (8)	−0.0191 (8)
O3W	0.0714 (12)	0.0408 (9)	0.0740 (12)	−0.0219 (8)	−0.0031 (10)	−0.0247 (8)

### Geometric parameters (Å, °)

**Table:**

Cd1—O1	2.3185 (13)	C4—H4	0.9300
Cd1—O1W	2.3336 (14)	C5—H5	0.9300
Cd1—N3	2.3513 (15)	C8—C9	1.395 (4)
Cd1—N1	2.3616 (14)	C8—H8	0.9300
Cd1—O3^i^	2.4049 (15)	C9—C10	1.351 (5)
Cd1—N2	2.4151 (16)	C9—H9	0.9300
Cd1—O4^i^	2.5189 (16)	C10—C11	1.400 (4)
O1—C6	1.256 (2)	C10—H10	0.9300
O1W—H1W1	0.8630	C11—C12	1.411 (3)
O1W—H2W1	0.8216	C11—C18	1.433 (4)
O2—C6	1.238 (2)	C12—C13	1.437 (3)
O3—C7	1.257 (2)	C13—C14	1.410 (3)
O3—Cd1^ii^	2.4049 (15)	C14—C15	1.399 (4)
O4—C7	1.238 (2)	C14—C19	1.422 (4)
O4—Cd1^ii^	2.5189 (16)	C15—C16	1.353 (4)
N1—C5	1.336 (2)	C15—H15	0.9300
N1—C1	1.341 (2)	C16—C17	1.396 (3)
N2—C8	1.324 (3)	C16—H16	0.9300
N2—C12	1.356 (3)	C17—H17	0.9300
N3—C17	1.322 (3)	C18—C19	1.331 (5)
N3—C13	1.353 (3)	C18—H18	0.9300
C1—C2	1.393 (2)	C19—H19	0.9300
C1—C6	1.526 (2)	O2W—H2W2	0.8556
C2—C3	1.389 (2)	O2W—H1W2	0.8277
C2—C7	1.501 (2)	O2W—H3W2	0.8415
C3—C4	1.377 (3)	O3W—H1W3	0.8306
C3—H3	0.9300	O3W—H2W3	0.8344
C4—C5	1.377 (3)	O3W—H3W3	0.8577
			
O1—Cd1—O1W	85.50 (5)	C4—C5—H5	118.9
O1—Cd1—N3	82.48 (5)	O2—C6—O1	125.52 (16)
O1W—Cd1—N3	87.94 (5)	O2—C6—C1	117.87 (15)
O1—Cd1—N1	70.02 (5)	O1—C6—C1	116.58 (15)
O1W—Cd1—N1	114.38 (5)	O4—C7—O3	122.84 (17)
N3—Cd1—N1	142.12 (5)	O4—C7—C2	119.52 (17)
O1—Cd1—O3^i^	139.09 (5)	O3—C7—C2	117.56 (16)
O1W—Cd1—O3^i^	78.30 (5)	N2—C8—C9	123.3 (3)
N3—Cd1—O3^i^	133.35 (5)	N2—C8—H8	118.4
N1—Cd1—O3^i^	82.89 (5)	C9—C8—H8	118.4
O1—Cd1—N2	91.39 (5)	C10—C9—C8	118.7 (3)
O1W—Cd1—N2	158.39 (6)	C10—C9—H9	120.6
N3—Cd1—N2	70.46 (6)	C8—C9—H9	120.6
N1—Cd1—N2	84.31 (6)	C9—C10—C11	120.5 (2)
O3^i^—Cd1—N2	116.44 (6)	C9—C10—H10	119.8
O1—Cd1—O4^i^	164.61 (5)	C11—C10—H10	119.8
O1W—Cd1—O4^i^	88.95 (5)	C10—C11—C12	117.3 (2)
N3—Cd1—O4^i^	82.98 (5)	C10—C11—C18	123.3 (2)
N1—Cd1—O4^i^	125.23 (5)	C12—C11—C18	119.3 (3)
O3^i^—Cd1—O4^i^	52.80 (5)	N2—C12—C11	121.9 (2)
N2—Cd1—O4^i^	88.50 (5)	N2—C12—C13	119.02 (17)
C6—O1—Cd1	118.77 (11)	C11—C12—C13	119.1 (2)
Cd1—O1W—H1W1	109.3	N3—C13—C14	121.7 (2)
Cd1—O1W—H2W1	103.1	N3—C13—C12	118.94 (17)
H1W1—O1W—H2W1	105.8	C14—C13—C12	119.32 (19)
C7—O3—Cd1^ii^	93.36 (11)	C15—C14—C13	117.7 (2)
C7—O4—Cd1^ii^	88.54 (12)	C15—C14—C19	122.6 (2)
C5—N1—C1	119.41 (15)	C13—C14—C19	119.7 (3)
C5—N1—Cd1	124.26 (12)	C16—C15—C14	119.9 (2)
C1—N1—Cd1	112.35 (11)	C16—C15—H15	120.0
C8—N2—C12	118.31 (19)	C14—C15—H15	120.0
C8—N2—Cd1	127.04 (16)	C15—C16—C17	118.9 (2)
C12—N2—Cd1	114.59 (13)	C15—C16—H16	120.5
C17—N3—C13	118.51 (18)	C17—C16—H16	120.5
C17—N3—Cd1	124.53 (14)	N3—C17—C16	123.2 (2)
C13—N3—Cd1	116.90 (13)	N3—C17—H17	118.4
N1—C1—C2	121.67 (15)	C16—C17—H17	118.4
N1—C1—C6	115.02 (14)	C19—C18—C11	121.4 (2)
C2—C1—C6	123.23 (15)	C19—C18—H18	119.3
C3—C2—C1	117.80 (16)	C11—C18—H18	119.3
C3—C2—C7	118.66 (15)	C18—C19—C14	121.2 (2)
C1—C2—C7	123.46 (15)	C18—C19—H19	119.4
C4—C3—C2	120.00 (17)	C14—C19—H19	119.4
C4—C3—H3	120.0	H2W2—O2W—H1W2	105.8
C2—C3—H3	120.0	H2W2—O2W—H3W2	101.4
C3—C4—C5	118.55 (16)	H1W2—O2W—H3W2	97.7
C3—C4—H4	120.7	H1W3—O3W—H2W3	106.6
C5—C4—H4	120.7	H1W3—O3W—H3W3	107.2
N1—C5—C4	122.22 (17)	H2W3—O3W—H3W3	109.5
N1—C5—H5	118.9		
			
O1W—Cd1—O1—C6	−131.03 (14)	Cd1—N1—C5—C4	−155.90 (15)
N3—Cd1—O1—C6	140.47 (14)	C3—C4—C5—N1	3.7 (3)
N1—Cd1—O1—C6	−13.05 (13)	Cd1—O1—C6—O2	−179.59 (14)
O3^i^—Cd1—O1—C6	−64.70 (16)	Cd1—O1—C6—C1	2.4 (2)
N2—Cd1—O1—C6	70.38 (14)	N1—C1—C6—O2	−158.83 (16)
O4^i^—Cd1—O1—C6	159.78 (16)	C2—C1—C6—O2	18.1 (2)
O1—Cd1—N1—C5	179.92 (16)	N1—C1—C6—O1	19.4 (2)
O1W—Cd1—N1—C5	−104.93 (15)	C2—C1—C6—O1	−163.76 (16)
N3—Cd1—N1—C5	133.86 (14)	Cd1^ii^—O4—C7—O3	15.87 (19)
O3^i^—Cd1—N1—C5	−31.25 (15)	Cd1^ii^—O4—C7—C2	−167.34 (15)
N2—Cd1—N1—C5	86.36 (15)	Cd1^ii^—O3—C7—O4	−16.7 (2)
O4^i^—Cd1—N1—C5	2.24 (17)	Cd1^ii^—O3—C7—C2	166.48 (13)
O1—Cd1—N1—C1	22.61 (11)	C3—C2—C7—O4	−119.5 (2)
O1W—Cd1—N1—C1	97.76 (12)	C1—C2—C7—O4	57.2 (3)
N3—Cd1—N1—C1	−23.44 (17)	C3—C2—C7—O3	57.5 (2)
O3^i^—Cd1—N1—C1	171.45 (13)	C1—C2—C7—O3	−125.86 (19)
N2—Cd1—N1—C1	−70.95 (12)	C12—N2—C8—C9	1.3 (4)
O4^i^—Cd1—N1—C1	−155.06 (11)	Cd1—N2—C8—C9	178.6 (2)
O1—Cd1—N2—C8	−98.04 (19)	N2—C8—C9—C10	−1.2 (4)
O1W—Cd1—N2—C8	−179.31 (17)	C8—C9—C10—C11	0.0 (5)
N3—Cd1—N2—C8	−179.6 (2)	C9—C10—C11—C12	0.8 (4)
N1—Cd1—N2—C8	−28.28 (19)	C9—C10—C11—C18	−178.2 (3)
O3^i^—Cd1—N2—C8	50.9 (2)	C8—N2—C12—C11	−0.4 (3)
O4^i^—Cd1—N2—C8	97.4 (2)	Cd1—N2—C12—C11	−178.00 (16)
O1—Cd1—N2—C12	79.33 (14)	C8—N2—C12—C13	179.3 (2)
O1W—Cd1—N2—C12	−1.9 (2)	Cd1—N2—C12—C13	1.7 (2)
N3—Cd1—N2—C12	−2.19 (13)	C10—C11—C12—N2	−0.6 (3)
N1—Cd1—N2—C12	149.10 (14)	C18—C11—C12—N2	178.4 (2)
O3^i^—Cd1—N2—C12	−131.76 (13)	C10—C11—C12—C13	179.6 (2)
O4^i^—Cd1—N2—C12	−85.28 (14)	C18—C11—C12—C13	−1.3 (3)
O1—Cd1—N3—C17	85.52 (17)	C17—N3—C13—C14	0.1 (3)
O1W—Cd1—N3—C17	−0.21 (17)	Cd1—N3—C13—C14	177.42 (15)
N1—Cd1—N3—C17	128.57 (16)	C17—N3—C13—C12	179.96 (18)
O3^i^—Cd1—N3—C17	−71.96 (19)	Cd1—N3—C13—C12	−2.7 (2)
N2—Cd1—N3—C17	179.69 (18)	N2—C12—C13—N3	0.6 (3)
O4^i^—Cd1—N3—C17	−89.41 (17)	C11—C12—C13—N3	−179.66 (18)
O1—Cd1—N3—C13	−91.62 (13)	N2—C12—C13—C14	−179.52 (18)
O1W—Cd1—N3—C13	−177.35 (13)	C11—C12—C13—C14	0.2 (3)
N1—Cd1—N3—C13	−48.57 (17)	N3—C13—C14—C15	0.6 (3)
O3^i^—Cd1—N3—C13	110.90 (14)	C12—C13—C14—C15	−179.3 (2)
N2—Cd1—N3—C13	2.56 (13)	N3—C13—C14—C19	−179.6 (2)
O4^i^—Cd1—N3—C13	93.46 (13)	C12—C13—C14—C19	0.6 (3)
C5—N1—C1—C2	−5.2 (3)	C13—C14—C15—C16	−0.8 (4)
Cd1—N1—C1—C2	153.32 (13)	C19—C14—C15—C16	179.4 (2)
C5—N1—C1—C6	171.73 (16)	C14—C15—C16—C17	0.3 (4)
Cd1—N1—C1—C6	−29.74 (17)	C13—N3—C17—C16	−0.6 (3)
N1—C1—C2—C3	6.6 (3)	Cd1—N3—C17—C16	−177.74 (18)
C6—C1—C2—C3	−170.04 (16)	C15—C16—C17—N3	0.5 (4)
N1—C1—C2—C7	−170.04 (16)	C10—C11—C18—C19	−179.3 (3)
C6—C1—C2—C7	13.3 (3)	C12—C11—C18—C19	1.7 (4)
C1—C2—C3—C4	−2.9 (3)	C11—C18—C19—C14	−1.0 (4)
C7—C2—C3—C4	173.96 (17)	C15—C14—C19—C18	179.6 (3)
C2—C3—C4—C5	−2.0 (3)	C13—C14—C19—C18	−0.2 (4)
C1—N1—C5—C4	−0.1 (3)		

Symmetry codes: (i) *x*+1, *y*, *z*; (ii) *x*−1, *y*, *z*.

### Hydrogen-bond geometry (Å, °)

**Table:**

*D*—H···*A*	*D*—H	H···*A*	*D*···*A*	*D*—H···*A*
O3W—H3W3···O2W	0.86	1.99	2.780 (3)	152
O3W—H2W3···O3W^iii^	0.83	1.98	2.795 (4)	164
O3W—H1W3···O1^iv^	0.83	2.06	2.860 (2)	161
O2W—H1W2···O2^iv^	0.83	2.02	2.840 (2)	173
O2W—H3W2···O2W^v^	0.84	1.93	2.748 (3)	163
O2W—H2W2···O3W	0.86	1.98	2.780 (3)	155
O1W—H2W1···O2^i^	0.82	1.97	2.7784 (19)	168
O1W—H1W1···O3^vi^	0.86	1.90	2.751 (2)	167

Symmetry codes: (iii) −*x*, −*y*+1, −*z*; (iv) −*x*, −*y*+1, −*z*+1; (v) −*x*+1, −*y*+1, −*z*; (i) *x*+1, *y*, *z*; (vi) −*x*, −*y*, −*z*+2.
